# Structural basis of the ligand binding and signaling mechanism of melatonin receptors

**DOI:** 10.1038/s41467-022-28111-3

**Published:** 2022-01-24

**Authors:** Qinggong Wang, Qiuyuan Lu, Qiong Guo, Maikun Teng, Qingguo Gong, Xu Li, Yang Du, Zheng Liu, Yuyong Tao

**Affiliations:** 1 Department of Clinical Laboratory, The First Affiliated Hospital of USTC, Ministry of Education Key Laboratory for Membraneless Organelles & Cellular Dynamics, Biomedical Sciences and Health Laboratory of Anhui Province, School of Life Sciences, Division of Life Sciences and Medicine, University of Science and Technology of China, 230027 Hefei, P.R. China; 2 Kobilka Institute of Innovative Drug Discovery, School of Life and Health Sciences, Chinese University of Hong Kong, 518172 Shenzhen, Guangdong China; 3 Ministry of Education Key Laboratory for Membraneless Organelles & Cellular Dynamics, Biomedical Sciences and Health Laboratory of Anhui Province, School of Life Sciences, Division of Life Sciences and Medicine, University of Science and Technology of China, 230027 Hefei, P.R. China

**Keywords:** Cryoelectron microscopy, G protein-coupled receptors

## Abstract

Melatonin receptors (MT_1_ and MT_2_ in humans) are family A G protein–coupled receptors that respond to the neurohormone melatonin to regulate circadian rhythm and sleep. Numerous efforts have been made to develop drugs targeting melatonin receptors for the treatment of insomnia, circadian rhythm disorder, and cancer. However, designing subtype-selective melatonergic drugs remains challenging. Here, we report the cryo-EM structures of the MT_1_–G_i_ signaling complex with 2-iodomelatonin and ramelteon and the MT_2_–G_i_ signaling complex with ramelteon. These structures, together with the reported functional data, reveal that although MT_1_ and MT_2_ possess highly similar orthosteric ligand-binding pockets, they also display distinctive features that could be targeted to design subtype-selective drugs. The unique structural motifs in MT_1_ and MT_2_ mediate structural rearrangements with a particularly wide opening on the cytoplasmic side. G_i_ is engaged in the receptor core shared by MT_1_ and MT_2_ and presents a conformation deviating from those in other G_i_ complexes. Together, our results provide new clues for designing melatonergic drugs and further insights into understanding the G protein coupling mechanism.

## Abstract (web summary)

Melatonin receptors (MT1 and MT2) are the targets for melatonin, the major neurohormone involved in circadian rhythm and sleep regulation. Here the authors describe the structures of 2-iodomelatonin and ramelteon bound MT1–Gi and MT2-Gi, revealing that MT1 and MT2 possess distinctive features within the ligand-binding pocket.

## Introduction

Melatonin receptors (MTs) are family A G protein-coupled receptors (GPCRs) expressed in the central nervous system (CNS) and peripheral tissues and are targets for melatonin, the major neurohormone involved in circadian rhythm and sleep regulation^1–3^. In vertebrates, melatonin (N-acetyl-5-methoxytryptamine) is mainly synthesized in the pineal gland and follows a circadian pattern synchronized to the dark phase of the natural light/dark cycle^4,5^. Modern life, such as worldwide travel, shift work, and artificial lighting, could disturb physiological melatonin production and cause sleep disorders, which was estimated to affect ~20% of the population in the US. As a result, melatonin is one of the most popular supplements for the treatment of insomnia and jetlag. In addition to entraining the sleep-wake rhythm^6^, the effects of melatonin via activation of MTs regulate other physiological processes, including modulating the cardiovascular system^7^ and buffering the immune system^8^. Emerging roles of MTs have also been confirmed in cancer protection^9,10^, bone formation^11^, glucose maintenance^12^, and neurodegenerative disorders^9^. Therefore, designing therapeutic agents targeting MTs has been continuously pursued in the drug discovery field^9^.

In humans, the MT family consists of two highly conserved members, termed MT_1_ and MT_2_, and they share 55% and 70% sequence similarity for the overall region and the transmembrane part, respectively. Both MT_1_ and MT_2_ predominantly signal through G_i/o_ proteins^13^, leading to inhibition of adenylyl cyclase and decreased intracellular concentrations of adenosine 3’,5’-cyclic monophosphate (cAMP). For other G-protein families, the exact coupling profile remains unclear. However, coupling of the MT_1_ receptor to G_q/11_ was consistently observed^14^. In the brain, MT_1_ is expressed in the Locus Coeruleus and lateral hypothalamus (REM areas)^15,16^, but MT_2_ is mainly located in the reticular thalamus (NREM area)^15,16^. The signaling variation and localization difference probably result in the distinct in vivo functions of MT_1_ and MT_2_^17^: MT_1_ is mainly implicated in the regulation of rapid eye movement (REM) phases of the vigilance state in sleep^18^; MT2 selectively increases non-REM (NREM) sleep^17–19^. In addition, single nucleotide polymorphisms (SNPs) revealed that MT_2_ is exclusively functionally relevant to type-2 diabetes (T2D)^12^. Given the different physiological roles of MT_1_ and MT_2_, obtaining selective ligands is highly desirable. However, most of the drugs on the market or under clinical evaluation, such as ramelteon^20^, tasimelteon^21^, and agomelatine^22^, are nonselective^23,24^. Currently, developing subtype-selective drugs, especially MT_1_-selective drugs, is very difficult. Moreover, the pharmacophores of the currently available melatonergic agonists are limited to very few types^25^. For example, an indene or naphthalene bioisostere group was repeatedly applied in melatonergic ligands^23–25^. Therefore, delineating the detailed configuration of the ligand-binding pocket of MTs is of great value for effective drug design. In this study, we determined the cryo-EM structures of active melatonin receptors engaged with the heterotrimeric G_i_ protein. These structures reveal distinctive features of the orthosteric pocket in active MTs and provide insights into MT activation and G protein-coupling preference.

## Result

### The overall structure of the MT_1_ and MT_2_ signaling complex

**Fig. 1:**
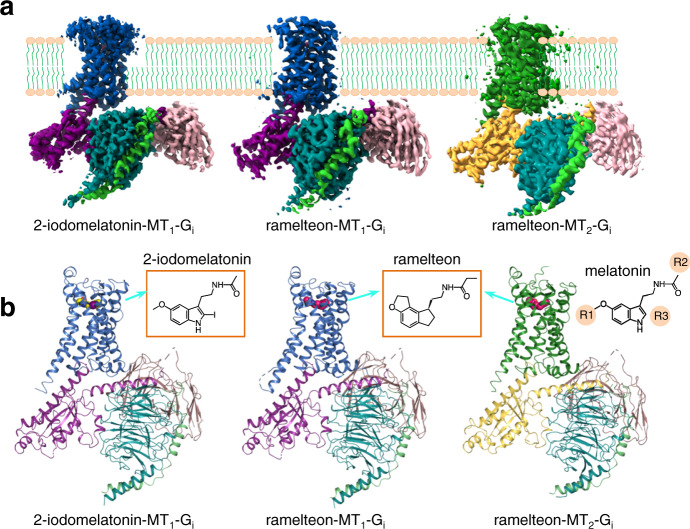
Overall structures of the MT_1_-G_i_ and MT_2_-G_i_ signaling complexes. **a** Cryo-EM density maps of the MT_1_-G_i_ and MT_2_-G_i_ complexes. The color code is as follows: MT_1_, blue; MT_2_, green; G_αi_ in MT_1_, purple; G_αi_ in MT_2_, yellow; G_β_, teal; G_γ_, light green; scFV16, violet. **b** Cryo-EM structures of the MT_1_-G_i_ and MT_2_-G_i_ complexes. Left, 2-iodomelatonin-bound MT_1_-G_i_; middle, ramelteon-bound MT_1_-G_i_; right, ramelteon-bound MT_2_-G_i_. The structures of the respective ligand and the melatonin molecule (right) are shown.

To obtain stable MT_1_-G_i_ protein complexes, the nonselective agonists 2-iodomelatonin^26^ and ramelteon^20^ exhibiting high potency and affinity for the melatonin receptor were used. The receptor and G protein were co-expressed in insect cells. The assembled complexes were then purified to homogeneity for single-particle cryo-EM studies. The structures of 2-iodomelatonin- and ramelteon-bound MT_1_-G_i_ complexes were determined with global resolutions of 3.1 and 3.3 Å, respectively (Fig. 1, Supplementary Figs. 1 and 2 and Supplementary Table 1). The relatively high-quality density maps of the two complexes together with the previously solved inactive structure of MT_1_ enabled us to confidently build atomic models composed of the ligands MT_1_, G_i_, and scFv16^27^. The majority of the side chains of MT_1_ and G_i_ protein residues are well defined in the structures (Supplementary Figs. 1 and 2). In addition to the protein part, an extra density located between the N-terminal portion of TM1 and TM7 was modeled as a cholesterol molecule.

The same strategy was then applied to prepare the agonist-bound MT_2_-G_i_ complex. Eventually, the reconstituted ramelteon-bound MT_2_-G_i_-scFv16 complex was subjected to cryo-EM data acquisition. After processing 120k particles from 3519 movies, an EM map of only 4.5 Å resolution was acquired. We speculated that improving the stability of the receptor might be helpful for obtaining high-resolution maps. Therefore, the three thermostable mutations, L108^ECL1^F/F129^3.41^W/C140^3.52^L, based on previous findings^28^, were chosen and introduced into MT_2_. These three residues were located far away from the ligand-binding pocket and the G protein-coupling interface^28^; as a result, their mutations minimally interfered with the function of the receptor in terms of ligand recognition and G protein coupling^28^. With this triple mutant complex of ramelteon-MT_2_-G_i_-scFv16, we obtained an EM density map at a global nominal resolution of 3.5 Å, which enabled us to model most portions of the receptor, the ramelteon molecule, the G_i_ protein, and the scFv16 (Fig. 1, Supplementary Fig. 3 and Supplementary Table 1).

The determined structures reveal that the overall assembly of MT_1_-G_i_ and MT_2_-G_i_ is similar to that of the most active GPCR–G-protein complexes (Fig. 1b). Both the ligands of 2-iodomelatonin and ramelteon used for complex reconstitution are bound at the orthosteric pockets of MT_1_ and MT_2_ (Fig. 1b). The 2-iodomelatonin-bound MT_1_-G_i_ complex shares a nearly identical overall structure with that of ramelteon-bound MT_1_-G_i_, with root-mean-square deviation (RMSD) values of 1 Å for the Cα atoms of the whole complexes and 0.8 Å for the Cα atoms of MT_1_ alone. The ramelteon-bound MT_2_-G_i_ complex adopts a conformation similar to that of MT_1_-G_i_, with an RMSD of 1.4 Å for the Cα atoms of the receptor part, reminiscent of the structural similarity of the two receptors in their inactive states. However, noticeable structural differences are also observed on the extracellular side and in the region involved in G-protein engagement, as discussed below.

### The orthosteric site and determinants of the ligand selectivity

**Fig. 2:**
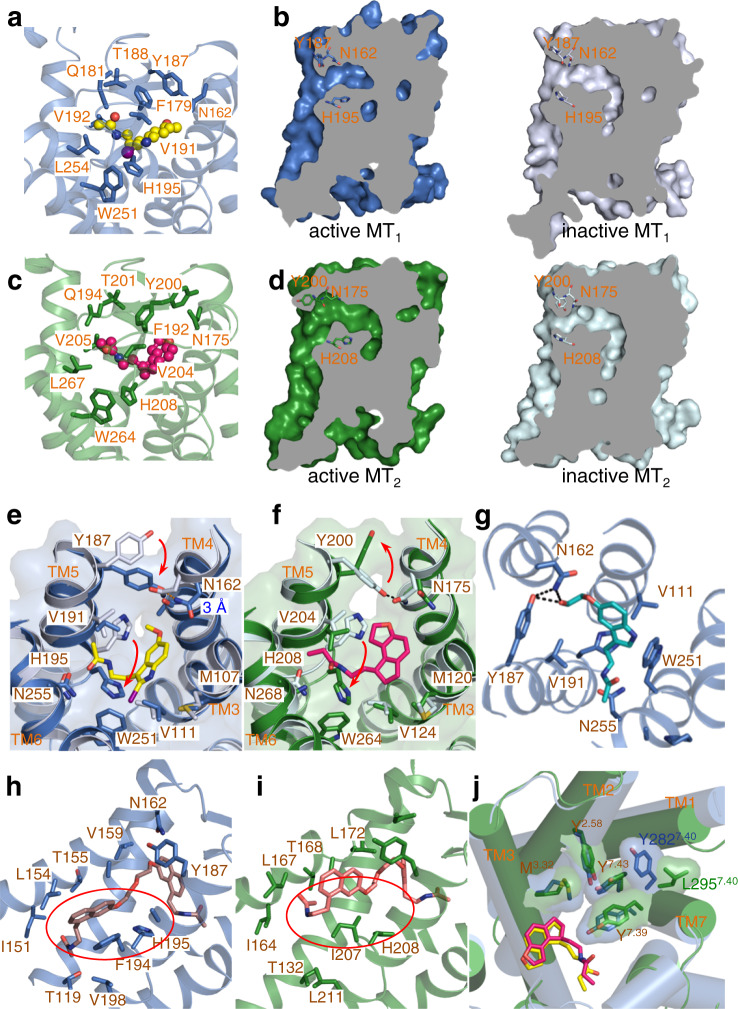
Ligand-binding pocket and selectivity determinants. **a** The ligand-binding pocket bound with the 2-iodomelatonin (yellow) in MT_1_ (blue). **b** Slab view showing the ligand access channel of MT_1_ in active (left) and inactive structures (right, light blue, PDB ID: 6ME4). **c** The ligand-binding pocket bound with the ramelteon (pink) in MT_2_ (green). **d** Slab view showing the ligand access channel of MT_2_ in active (left) and inactive structures (right, light cyan, PDB ID: 6ME9). **e** Comparison of the residues from the active (blue) and inactive (light blue) ligand-binding pockets of MT_1_. Red arrows indicate remarkable conformational changes. The distance between the two atoms forming the N162/Y187 hydrogen bond is 3 Å. **f** Comparison of the residues from the active (green) and inactive (light cyan) ligand-binding pockets of MT_2_. Red arrows indicate remarkable conformational changes. **g** 5-HEAT (cyan) docked in the active MT_1_. Key interacting residues from the pocket are shown as sticks. **h** CTL 01-05-B-A05 (salmon) docked in the active MT_1_. The red circle indicates the hydrophobic packing between F194^5.45^ and the naphthalene group. **i** CTL 01-05-B-A05 (salmon) docked in the active MT_2_. The red circle highlights the incompatible packing between I207^5.45^ and the naphthalene group. **j** Comparison of the subpockets from active MT_1_ (blue) and MT_2_ (green) bound with ramelteon (red in MT_1_, yellow in MT_2_). Key different residues are shown as sticks.

In both the MT_1_ and MT_2_ structures, the ligand of 2-iodomelatonin or ramelteon binds at the orthosteric pocket constituted by TM3, TM5, TM6, TM7, and ECL2 (Fig. 2a, c). The binding pose of the ramelteon in active MT_1_ or MT_2_ is superimposable with those in inactive structures (Supplementary Fig. 4). However, the binding pose of the 2-iodomelatonin in the active structure is slightly different from that in the inactive structure, especially in the region of the alkylamide tail, which moves closer to the toggle switch residue W^6.48^ in active MT_1_ (Supplementary Fig. 4). ECL2 sits on the top of the pocket and completely blocks ligand access from the extracellular side in both structures (Fig. 2b, d). The only entrance to the orthosteric-binding site in the active state is the previously identified lateral channel between TM4 and TM5 (Fig. 2b, d). Comparison of the orthosteric pocket from active and inactive structures revealed remarkable differences: the pocket in the active structures is more constricted in the central part, but it extends to connect with a wide “longitudinal channel” formed by TM3, TM4, and TM5 (Fig. 2b, d). Inspection of the residues around the pockets reveals that while those surrounding the iodine group and alkylamide tail (named R^3^ position in melatonin, Fig. 1) align well, several residues lining the solvent channel present very different conformations (Fig. 2e, f), which results in the varied configuration of the active pocket. Specifically, the aromatic residue Y187^5.38^ in MT_1_ rotates from the solvent-facing conformation in the inactive structure toward TM4 and forms a hydrogen bond with N162^4.60^ in the active structure (Fig. 2e). This hydrogen bond further reduces the diameter of the ligand entrance and could restrict the release of the bound agonist, as the Y187^5.38^A mutation led to a fast ligand dissociation rate^27,28^. In addition, the N162^4.60^A mutation disrupted the function of MT_1_, again highlighting the functional importance of this hydrogen pair in MT_1_ activation^27,28^ (Supplementary Fig. 5). In MT_2_, the corresponding pair N175^4.60^ and Y200^5.38^ also experienced conformational changes during transmission from the inactive to the active state (Fig. 2f). However, they take an opposite manner, and the hydrogen bond formed in the inactive structure disappears in the active structure (Fig. 2f). The absence of this hydrogen bond indicates that MT_2_ does not need an MT_1_-like entrance-restricting hydrogen bond for activation, correlating well with the previous finding that the N175^4.60^ A mutation showed no functional impairment^27,28^ (Supplementary Fig. 5). Notably, to facilitate the conformational changes of this N^4.60^-Y^5.38^ pair, a conserved proline (P^4.59^) is located close to N^4.60^ in MT_1_ and MT_2_, and mutation of P^4.59^ disrupts the ligand-binding activity of MT_2_^29^.

In addition to the conformational changes in the N^4.60^-Y^5.38^ pair, residue H^5.46^ (H195^5.46^ in MT_1_ and H208^5.46^ in MT_2_) (Fig. 2e, f), which is positioned two helical turns below Y^5.38^, displays the most striking difference in the pockets from the inactive and active structures (Fig. 2e, f). In the inactive structures, H^5.46^ packs against TM4 and stays far away from the bound ligand (Fig. 2e, f). However, in the active structures, it undergoes a 2.4 Å inward displacement and adopts a flipped rotamer of the side chain, creating van der Waals interactions with the alkylamide tail of the bound ligand and the toggle switch residue W^6.48^ (W251^6.48^ in MT_1_ and W264^6.48^ in MT_2_) (Fig. 2e, f). This rearranged conformation of H^5.46^ explains the previous observation that the H208^5.46^A mutation affected MT_2_ function^28,30^. Interestingly, although H^5.46^ undergoes similar conformational changes in MT_1_ and MT_2_, its functional importance seems to be different in the two receptors, as the mutation of H208^5.46^ (MT_2_) only moderately impaired the function of MT_2_, but the H195^5.46^A (MT_1_) mutation caused a deleterious effect, as reflected by the low expression level of the mutant^27,28^. Owing to the movement of H^5.46^, additional space adjacent to the methoxy group of 2-iodomelatonin (named R^1^ position in melatonin) is produced, resulting in the formation of the abovementioned “longitudinal channel” (Fig. 2b, d). We then docked two representative ligands that possess moderate MT_1_ selectivity, 5-hydroxyethoxy-N-acetyltryptamine (5-HEAT)^31^ and CTL 01-05-B-A05^27^, to both receptors (Supplementary Fig. 6). Compared to melatonin, both 5-HEAT and CTL 01-05-B-A05 have substituents at the R1 position. In MT_1_, 5-HEAT occupied a position superimposable with that of the bound 2-iodomelatonin by forming hydrogen bonds with N162^4.60^ and Y187^5.38^ (Fig. 2g). Thus, the molecular structure of 5-HEAT is probably compatible with the pose of the active pocket required to activate MT_1_, resulting in 5-HEAT being an agonist of MT_1_. In contrast, as an antagonist of MT_2_, 5-HEAT did not take a plausible docking position in the observed active pocket of MT_2_, probably due to the varied conformations of N175^4.60^ and Y200^5.38^. 5-HEAT probably utilizes an induced-fit model for binding MT_2,_ and the resultant configuration of the MT_2_ pocket probably loses the ability to induce intracellular structural rearrangement. CTL 01-05-B-A05 adopts an extended conformation in MT_1_ with the second naphthalene group packing against F194^5.45^ (Fig. 2h). However, CTL 01-05-B-A05 binding to MT_2_ was suboptimal, as the stacking interaction was disrupted by the precluding side chain of I207^5.45^ at the corresponding position (Fig. 2i). Notably, based on our results, it is worth further optimizing the bitopic ligand to obtain more selective MT_1_ agonists. For example, introducing novel substituents into the second unit for optimal fitting with the “longitudinal channel” will be a promising strategy.

Although the pockets from inactive MT_1_ and MT_2_ showed only subtle conformational differences in the region around the R^3^ group of the ligand (termed subpocket), they became more discriminative in the active structures. MT_1_ has a tyrosine at the position of 7.40 (Y282^7.40^), while the corresponding residue in MT_2_ is a leucine (L295^7.40^) (Fig. 2j). Packing of Y282^7.40^ against TM1 pushes the two adjacent residues Y281^7.39^ and Y285^7.43^ closer to the core of the pocket than the equivalent residues Y294^7.39^ and Y298^7.43^ in MT_2_ (Fig. 2j). As a result, MT_2_ has a larger subpocket, allowing the accommodation of ligands with bulky R^3^ substituents, consistent with the molecular structures of most MT_2_ selective agonists^23,30^. In summary, the structural and functional data revealed that although the ligand-binding pockets in MT_1_ and MT_2_ share similarities, they also present distinct features. The unique configuration of the N^4.60^-Y^5.38^-H^5.46^ motif and the “longitudinal channel” in MT_1_ and the larger subpocket in MT_2_ could be targeted to design MT subtype-selective drugs.

### The active conformation of MT_1_ and MT_2_

**Fig. 3:**
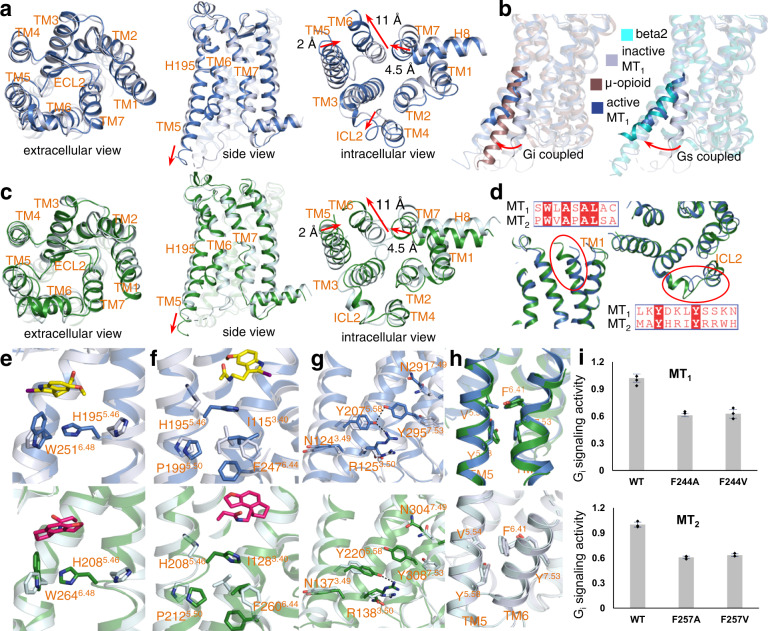
MT_1_ and MT_2_ activation. **a** Structural comparison of the active (blue) and inactive (light blue) MT_1_. Three views are presented. **b** Comparison of the conformation of TM6 from MT_1_ with that from the G_i_-coupled receptor μ-opioid (left, PDB ID: 6DDE) receptor and G_s_-coupled receptor beta2 (right, PDB ID: 3SN6). **c** Structural comparison of active (green) and inactive (light green) MT_2_. Three views are presented. **d** Comparison of TM1 (left) and ICL2 (right) from MT_1_ and MT_2_. **e**–**g** Conformational changes of the key residues and motifs during receptor activation in MT_1_ (above) and MT_2_ (below). **h** The conformations of F^6.41^ in active (above) and inactive (below) MT_1_ and MT_2_ structures. **i** The Gi signaling ability of the F^6.41^ mutant of MT_1_ and MT_2_. Data were normalized to that of the wild-type receptor and presented as the means ± standard error of three replicates.

MT_1_ from the G_i_-coupled structures adopts an active conformation with the characteristic of TM6 outward displacement, similar to the other active GPCRs. Comparison of the active and inactive structures revealed that MT_1_ maintains a similar conformation on the extracellular side, probably because both of them were stabilized by the agonist (Fig. 3a). In contrast, extensive structural rearrangements occur at the intracellular part (Fig. 3a). Among them, the 11 Å outward displacement of TM6 is most striking (Fig. 3a). In general, the outward displacement angle of TM6 is smaller in Gi/o-coupled GPCRs than in Gs-coupled GPCRs. However, MT_1_ TM6 has a relatively large displacement, reaching the extent observed in G_s_-coupled GPCRs^32^ (Fig. 3b and Supplementary Fig. 7). This unusual movement of TM6 is coordinated by the unique structural motifs in MTs, as discussed below. Accompanying TM6 movement, TM7 also undergoes conformational changes, and it takes 4.5 Å movement toward TM6 (Fig. 3a). Induced by the reorientation of H195^5.46^, TM5 also shifts 2 Å closer to TM6 (Fig. 3a). Moreover, its cytoplasmic end, which mediates interactions with the Ras-like domain of G_αs_, is extended by two additional helical turns in the active structure (Fig. 3a). Last, residue C130^3.55^ bends the TM3 helix and reshapes the trajectory of ICL2 (Fig. 3a and Supplementary Fig. 8). This repositioning of TM3 and ICL2 is necessary for G_i_ accommodation; otherwise, they would make a severe steric clash with the αN of G_i_ (Supplementary Fig. 8). Consistently, the MT_1_ C130^3.55^ mutation led to a decreased G-protein activation ability^33^.

As expected, MT_2_ also undergoes structural rearrangements in the regions of TM5, TM6, and TM7, resulting in an active conformation almost identical to that of MT_1_ (Fig. 3c), consistent with their sequence homology and the structural resemblance between the inactive states. Conformational differences between active MT_1_ and MT_2_ were only observed in the extracellular end of TM1 and ICL2, where minimal sequence conservation exists (Fig. 3d). As mentioned above, the bulky Y282^7.40^ in MT_1_, which is linked to the slight differences between the ligand-binding pockets, pushes TM1 more outward from the TM bundle (Fig. 3d). ICL2 in MT_2_ adopts a different trajectory than that in MT_1_ (Fig. 3d). The varied ICL2 in terms of sequence composition and spatial localization probably confers the slightly different G protein-coupling profiles of MT_1_ and MT_2_, as discussed below.

Comparison of the conserved motifs in MT_1_ and MT_2_ with those in other class A GPCRs reveals that they possess common as well as unique features. The toggle switch W^6.48^ (W251^6.48^ in MT_1_ and W264^6.48^ in MT_2_) makes direct contact with the alkylamide tail of the ligand (Fig. 3e), accounting for the linkage between ligand binding and receptor activation. Based on the established activation mechanism of GPCRs, the toggle switch residue often undergoes conformational changes upon agonist binding. However, how MT_1_ and MT_2_ W^6.48^ experience conformational changes remain unknown, as the state of W^6.48^ from an inactive structure is lacking. W^6.48^ was mutated to Phe in the previous inactive structures for stability improvement. The triadmotif P^5.50^-I^3.40^-F^6.44^, which is located close to the toggle switch, also experiences classical rearrangement (Fig. 3f). Interestingly, the repositioned H195^5.46^ (H208^5.46^ in MT_2_) in active MT_1_ sits right between the ligand and I^3.40^ from the triadmotif and simultaneously forms interactions with the ligand and I^3.40^ (Fig. 3e, f). Therefore, H195^5.46^ might cooperate with W^6.48^ to link ligand binding with receptor activation: agonistic ligand binding to the orthosteric site induces conformational changes in H195^5.46^ and W^6.48^ by forming direct interactions; H195^5.46^ and W^6.48^ then propagate the signals to the P^5.50^-I^3.40^-F^6.44^ triadmotif located right below; and the rearranged triadmotif finally drives the movement of TM5 and TM6, leading to receptor activation. On the intracellular side, the typical N^7.49^P^7.50^xxY^7.53^ motif in TM7 is replaced with N^7.49^A^7.50^xxY^7.53^ in MT_1_ and MT_2_. This unusual motif is functionally essential, as the converted N^7.49^P^7.50^xxY^7.53^ motif led to a loss of function in MT_2_^29^. Similarly, the canonical D^3.49^R^3.50^Y^3.51^ motif was changed to N^3.49^R^3.50^Y^3.51^ in MT_1_ and MT_2_. Y^5.58^ in TM5 forms hydrogen bonds with both R^3.49^ and Y^7.53^, acting to tether the cytoplasmic ends of TM3, TM5, and TM7 (Fig. 3g). Notably, the commonly observed small hydrophobic residue V^6.41^ in most class A GPCRs is replaced by F^6.41^ in MT_1_ and MT_2_. In the inactive structures, F^6.41^ points outside and packs against V^5.54^. However, in the active structures, it flips close to Y^7.53^ and stacks with Y^5.58^ (Fig. 3h). This special hydrophobic core induces the particularly wide opening of TM6. Mutation of F^6.41^ into valine or alanine crippled the signaling activity of MT_1_ and MT_2_, demonstrating that a large cytoplasmic cavity is required for G_i_ engagement in MT_1_ and MT_2_ (Fig. 3i). In summary, through structural rearrangements mediated by classical and unique microswitches, MT_1_ and MT_2_ adopt a similar active conformation with a wide opening in the cytoplasmic core.

### Interactions between MT_1_, MT_2,_ and G_i_

**Fig. 4:**
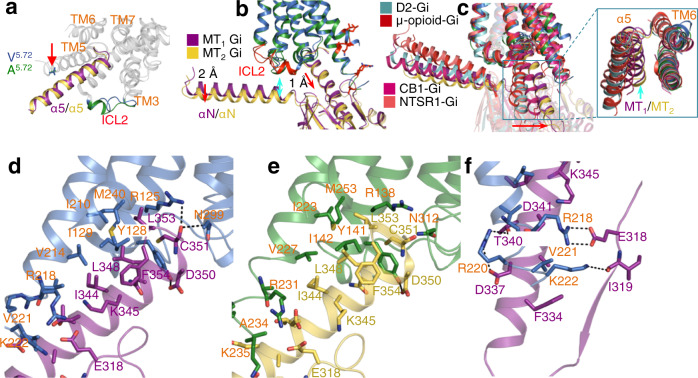
The G_i_ conformations and interfaces in MT_1_-G_i_ and MT_2_-G_i_ complexes. **a** Comparison of the receptor core (gray) engaging the α5 helix in MT_1_-G_i_ (purple) and MT_2_-G_i_ (yellow) complexes. The indicated (red arrow) residue 5.72 is the only different residue (V^5.72^ in MT_1_ and A^5.72^ in MT_2_). **b** The conformations of the G_i_ bound to MT_1_ and MT_2_. Displacements of αN and α5 are highlighted with red arrows. αN is located far from ICL2, indicated by the cyan arrow. **c** The orientations of the α5 helices in MT_1_, MT_2_, and other G_i_-coupled receptors. **d, e** α5 helix-mediated interactions in MT_1_-G_i_ (**d**) and MT_2_-G_i_ (**e**) complexes. **f** The polar interactions in the MT_1_-G_i_ interface. Hydrogen bonds are highlighted with dashes.

The interactions between MT_1_, MT_2_, and G_i_ are mainly mediated by the α5 helix of the G_αi_ subunit, and the receptor cores comprise TM3 and TM5-7 from MT_1_ and MT_2_ (Fig. 4a). Although ICL2 forms hydrophobic or polar interactions with the G protein in most GPCRs, it mediates minimal contact with α5 and αN in G_i_ bound to MT_1_ and MT_2_ (Fig. 4b). As a result, the G_i_ interfaces in MT_1_ and MT_2_ are almost indistinguishable (Fig. 4a). Interestingly, even though MT_1_ and MT_2_ employ a nearly identical set of residues for accommodating G_i_, the bound G_i_ still displays slightly different conformations (Fig. 4b). α5 and αN in MT_2_-G_i_ have outward displacements of 1 and 2 Å, respectively, compared to MT_1_-G_i_ (Fig. 4b). This conformational difference may be attributed to ICL2. The varied ICL2 in MT_1_ and MT_2_ might pose different impacts during G_i_ engagement, which results in the observed G_i_ conformations. Another possibility is that these two structures represent different conformational states during G_i_ binding to MT_1_ and MT_2_. Although the MT_1_–G_i_ and MT_2_–G_i_ complexes adopt a commonly observed overall assembly mode, they display distinct features as well. When aligned on the receptors, the G_i_ bound to MT_1_ and MT_2_ show a relative orientation completely different from those presented by the G_i_ in complex with the μ-opioid^34^, dopamine D2^35^, CB1 cannabinoid^36^, and NTSR1 neurotensin receptors^37^ (Fig. 4c). The α5 helix in MT_1_–G_i_ and MT_2_–G_i_ undergoes significant rotational and translational movement away from TM3 and forms tight packing interactions with TM5 in an antiparallel manner (Fig. 4c). In contrast, owing to the large outward displacement of TM6, much fewer contacts are formed between TM6 and G_αi_ in the MT_1_-G_i_ and MT_2_-G_i_ complexes (Fig. 4c).

Hydrophobic contacts dominate the part formed between the C-terminus of α5 helix and the receptor core, exemplified by the I344^G.H5.16^, L348^G.H5.20^, L353^G.H5.25^, and C351^G.H5.23^ interacting with the residues from TM3, TM5, and TM6 (Fig. 4d, e). Remarkably, the involved Y^3.53^ in MT_1_ and MT_2_ frequently appears as alanine in most class A GPCRs. In MT_1_-G_i_ and MT_2_-G_i_, the bulky side chain of Y^3.53^ probably contributes to establishing the special position of G_i_ by restricting the α5 helix from moving away from TM5 (Fig. 4d, e). Indeed, mutation of Y^3.53^ of MT_2_ caused impaired G_i_ signaling ability^38^. In MT_1_, V221^5.72^ mediates another hydrophobic contact by burying its side chain into a hydrophobic pocket formed by Y320^G.S6.01^, F334^G.H5.06^, and A337^G.H5.09^ (Fig. 4f). Polar interactions are also observed in the MT-G_i_ interface and are mainly contributed by the three basic residues located in the cytoplasmic end of TM5: R^5.69^ forms a salt bridge with E318^G.h4s6.12^ from G_αi_; R^5.71^ (K^5.71^ in MT_2_) and K^5.73^ forms a hydrogen bond with T340^G.H5.12^ and the backbone carbonyl of I319^G.S6.01^, respectively (Fig. 4f). Due to the α5 movement away from TM3, most of the previously observed interactions between ICL2 and αN disappear in MT_1_-G_i_ and MT_2_-G_i_ complexes. For example, the well-known interaction mediated by the side chain of residue 34.51 from ICL2 and the hydrophobic cavity formed by the α5, αN, and β2-β3 loop of G_αi_ is not observed in the MT_1_-G_i_ and MT_2_-G_i_ complexes.

Although MT_1_ and MT_2_ share almost identical residues in the receptor core, they still possess varied G protein-coupling abilities. MT_2_ exclusively couples to G_i/o_, but MT_1_ could also couple to G_q/11_. Therefore, MT_1_ should possess a special region in addition to the observed G_i_ interface to support G_q/11_ engagement. As mentioned above, ICL2, which is involved in the G-protein association in most GPCR–G-protein complexes, indeed displays obvious sequence and conformational differences in MT_1_ and MT_2_ (Fig. 4a). Thus, ICL2 in MT_1_ probably participates in the constitution of a unique interface, at which G_q/11_ binds and produces a resultant conformation different from that of the observed G_i_. In fact, this phenomenon has been previously observed for the two closely related receptors M1 and M2 muscarinic acetylcholine receptors: G_11_ and G_i_ were bound at different regions in the receptors, and their conformations were also different^39^. Altogether, MT_1_ and MT_2_ share a unique G_i_ interface featuring the antiparallel packing of TM5 against the α5 of G_i_, and the varied ICL2 probably confers the additional subtype G protein-coupling ability of MT_1_.

### Disease-related mutations in MT_2_ receptor

MT_2_ loss-of-function has been linked to type-2 diabetes (T2D)^12,38^. Of the mutants associated with T2D, nearly half impair G_i_ signaling ability. When we mapped these affected sites onto the MT_2_-G_i_ structure, we observed clustering of residues around the G_i_ interface (Supplementary Fig. 9). Based on their positions, these mutations can be classified into three categories. The first category mutations are in the GPCR key functional motif, such as R138^3.50^H/L/C from the DRY motif and Y308^7.53^S of the NAxxY motif. The second category mutations are positioned in the G_i_ interface, such as Y141^3.53^F and A234^5.72^T. The third category of mutations is located away from the above-identified functional region. They seem to impair G_i_ binding by influencing the conformation of the cytoplasmic core. For example, R222^5.60^, which sits at the cytoplasmic edge of TM5, might contact the phosphate group from the membrane and contribute to shaping the conformation of TM5. Its mutation to histidine probably disrupts this interaction and leads to a varied configuration of TM5 without the G_i_ coupling ability.

## Discussion

Melatonin receptors have always been regarded as drug targets for developing therapeutics to treat insomnia, circadian rhythm and mood disorders, and cancer. Efforts to identify novel ligands with exceptional properties have been constantly made in the past four decades. However, only a limited number of chemotypes have been discovered for melatonergic ligands. The reason partially lies in the lack of structures providing information on the orthosteric ligand pocket. Indeed, immediately following the recent structural determination of MT_1_ and MT_2_, new melatonergic ligands possessing novel chemotypes were successfully discovered by screening the ZINC database^40,41^. Therefore, uncovering the features of the ligand-binding site of melatonin receptors has valuable pharmacological significance. In this study, we solved the structures of MT_1_ and MT_2_ coupled to G_i_ protein, the form representing the active state during receptor signaling. Interestingly, we found that the conserved H^5.46^, N^4.60,^ and Y^5.38^ positioned around the ligand pocket adopt conformations different from those captured in the inactive state. These varied conformations result in a quite different ligand-binding site and the associated channel to the site. While our manuscript is in submission, Okamoto et al. report the structure of ramelteon-MT_1_-G_i_ with varied conformations of H^5.46^, N^4.60^, and Y^5.38^ ^42^. However, an inspection of their data revealed that the quality of their density map was poor and that the model was incorrectly built, especially in the regions of TM5, TM6, and ICL2 and for the residues of H^5.46^, N^4.60^, and Y^5.38^ (Supplementary Fig. 10). Moreover, their map supports the model built here (Supplementary Fig. 10). Together, the distinctive feature of the MT_1_ ligand site revealed here provides an unrevealed clue for designing novel ligands with MT_1_ selectivity in the future. To date, the observed ligand-binding pocket in all solved structures is shaped with agonists sharing a melatonin-like chemotype core. However, when binding to the recently identified ligands with a new chemotype, reorganization of the pocket with remarkable differences from the current pocket probably would occur. Such plasticity in the ligand-binding site is also observed in other receptors, such as in the CB1 receptor^36^. Therefore, further delineating the pocket bound with various ligands will potentially drive a more efficient structure-based drug design.

One of the fundamental questions in the GPCR field is the G protein-coupling selectivity. To date, no general rule guiding GPCR–G-protein assembly has been concluded. For example, although muscarinic receptors share high sequence homology, they display different G protein-coupling profiles^39^. Similarly, MT_1_ shares high sequence identity with MT_2_, but it couples more G-protein subtypes. Here, we revealed that MT_1_ and MT_2_ utilize a nearly identical set of residues to engage G_i_. The combined sequence and conformation of the receptor core result in a unique G_i_ binding mode, consistent with the observation of divergent G_i_ conformations in the reported structures. Although ICL2 participates in the interactions with G_i_ in almost all the reported structures, it does not play a similar role in MT_1_ and MT_2_. However, ICL2 is the only cytoplasmic region where clear differences were observed between MT_1_ and MT_2_. Therefore, the ICL2 of MT_1_ probably confers selective MT_1_ coupling to G_q/11_ by generating a special interface different from that used for G_i_. Altogether, our study reveals the structural basis of G protein coupling to melatonin receptors and provides new clues for MT_1_ and MT_2_ selective drug design.

## Methods

### Constructs and complex expression

The coding sequence of wild-type human MTNR_1_A (MT_1_) or modified human MTNR_1_B (MT_2_) was cloned into the pFastbac A vector (Thermo Fisher Cat# 10360014) with a hemagglutinin (HA) signal peptide followed by a FLAG tag at the N-terminus and a hexa-histidine tag at the C-terminus. Three mutations (L108F, F129 W, and C140 L) were introduced into MT_2_ to improve the protein behavior. Dominant-negative Gαi1 (DNGαi1) with mutations (G203A and A326S)^43^ was cloned into the pFastbac A vector, and Gβ_1_γ_2_ was cloned into the pFastbac Dual vector. MT_1,_ MT_2_, DNGαi1, and Gβ_1_γ_2_ were co-expressed in *Spodoptera frugiperda Sf9* cells (Invitrogen Cat# A35243) using the Bac-to-Bac baculovirus expression system (Thermo Fisher Cat# 10360014). Cells were infected at a density of 4 × 10^6^ cells per ml with baculoviruses expressing MT_1_ or MT_2_, DNGαi1, and Gβ_1_γ_2_ at a ratio of 30:5:1. The infected cells were cultured at 27 °C for 48 h, collected by centrifugation at 1000 × *g* for 10 min, and then stored at −80 °C for future use.

### Complex formation and purification

To obtain the GPCR–G_i_ complex, cell pellets were thawed and suspended in lysis buffer (10 mM HEPES, pH 7.5, 0.5 mM EDTA) supplemented with 5 μM ramelteon (agonist, MCE Cat# HY-A0014) or 2-iodomelatonin (agonist, MCE Cat# HY-101176) and then rotated at 4 °C for 60 min to induce the formation of the complexes. Cell membranes were collected and homogenized in solubilization buffer buffer containing 20 mM HEPES pH 7.5, 100 mM NaCl, 5 µM agonist, 10% glycerol, 1% (w/v) n-Dodecyl-B-D-Maltoside (DDM, Anatrace Cat# D310), 0.1% (w/v) cholesteryl hemisuccinate (CHS, Sigma Cat#C6512), 0.2 µg ml^−1^ leupeptin (Sigma–Aldrich Cat# L5793), 100 µg ml^−1^ benzamidine (Sigma–Aldrich Cat# 12072), 10 mM MgCl_2_, 5 mM CaCl_2_, 1 mM MnCl_2_, 100 μU ml^−1^ lambda phosphatase (NEB Cat# P0753S), and 25 μU ml^−1^ apyrase (NEB Cat# M0398S), and then incubated at 4 °C for 90 min. The supernatant was isolated by centrifugation at 28,000 × *g* for 60 min and incubated with the Anti-FLAG M1 affinity resin (M1 resin, Sigma–Aldrich Cat# A4596) at 4 °C for 90 min. M1 resin was then collected and washed with the wash buffer containing 20 mM HEPES pH 7.5, 100 mM NaCl, 5 µM agonist, 0.1% DDM, 0.01% CHS, and 2 mM CaCl_2_. Buffer exchange from DDM buffer to lauryl maltose neopentyl glycol (LMNG, Anatrace, Cat# 4216588) buffer was performed in a stepwise manner. After buffer exchange, the M1 resin was further washed with LMNG buffer containing 20 mM HEPES pH 7.5, 100 mM NaCl, 5 µM agonist, 0.01% (w/v) LMNG, 0.001% (w/v) CHS, and 2 mM CaCl_2_. The complex was eluted with elution buffer containing 20 mM HEPES pH 7.5, 100 mM NaCl, 5 µM agonist, 0.00075% (w/v) LMNG, 0.00025% (w/v) glycol-diosgenin (GDN, Anatrace Cat#GDN101), 0.0001% CHS, 5 µM agonist, 5 mM EDTA, and 200 µg ml^−1^ synthesized Flag peptide. The eluted complex was concentrated and incubated with scFv16^44^ at a molar ratio of 1:1.5 for 60 min on ice. The complex was further purified by size-exclusion chromatography using a Superdex 200 Increase 10/300 column (GE Healthcare) pre-equilibrated with buffer containing 20 mM HEPES, pH 7.5, 100 mM NaCl, 0.00075% (w/v) LMNG, 0.00025% (w/v) GDN, 0.0001% (w/v) CHS, and 5 μM agonist. The complex fractions were collected and concentrated to 5 mg/ml using a 100-kDa molecular weight cutoff concentrator (Millipore) for electron microscopy experiments.

### Cryo-grid preparation and EM data collection

To prepare the cryo-EM sample, 3 μl purified complex was applied onto the amorphous alloy film grid (M024-Au300-R12/13)^45^ or 100 Holey Carbon film (Au, 300 mesh, N1-C14nAu30-01), glow-discharged by the easiGlow™ Glow Discharge Cleaning System (PELCO, USA) at 15 mA for 45 s. At 4 °C and 95% humidity, the sample was waited for 3 s and blotted for 2 s. Grids were plunge-frozen in liquid ethane cooled by liquid nitrogen using Vitrobot Mark IV (Thermo Fisher Scientific) and then stored in liquid nitrogen until checked. Movies were collected on a 300 kV Titan Krios Gi3 microscope (Thermo Fisher Scientific FEI, the Kobillka Cryo-EM Center of the Chinese University of Hong Kong, Shenzhen). The raw movies were recorded by a Gatan K3 BioQuantum camera at a magnification of 105,000, and the pixel size was 0.83 Å. Inelastically scattered electrons were excluded by a GIF Quantum energy filter (Gatan, USA) using a slit width of 20 eV. The movie stacks were acquired with the defocus range of −1.1 to −2.0 micron with a total exposure time of 2.5 s fragmented into 50 frames (0.05 s/frame) and with a dose rate of 21.2 e/pixel/s. Automated single-particle data acquisition was performed using SerialEM 3.7.

### Image processing and 3D reconstructions

Image processing of the 2-iodomelatonin-MT_1_-G_i_-scFv16 complex was conducted as previously described^46,47^. Briefly, data binned four times were used for micrograph screening and particle picking, and data binned two times were used for particle screening and classification. The particles after initial cleaning were subjected to particle extraction from the originally cleaned micrograph, and the resultant dataset was used for final cleaning and reconstruction. Specifically, raw movie frames were aligned with MotionCor2 using a 9 × 7 patch, and the contrast transfer function (CTF) parameters were estimated using Gctf and ctf in JSPR^48^. Only the micrographs with consistent CTF values, including defocus and astigmatism parameters, were kept for subsequent image processing. This process kept 3513 micrographs from 3762 raw movies. Templates for particle selection were generated by projecting the 3D volume of the V2R-Gs complex^49^. The 3,578,225 particles picked from template picking were subjected to 2 rounds of 2D classification, reducing their size to 1,131,706 and then reducing to 773,176 by 3D classification. After several rounds of ab initio refinement, the particles kept at 379,512 were subjected to nonuniform refinement for a 3.05 angstrom reconstruction. The image parameters were converted back and to Relion^50^ and cryoSPARC^51^ by use of the pyem package^52^. For the ramelteon-MT_1_-G_i_-scFV16 complex, dose-fractionated image stacks of the collected movies were subjected to beam-induced motion correction using MotionCor2.1^53^, and the CTF parameters for each micrograph were determined by Gctf^54^. The 2-iodomelatonin-MT_1_-G_i_-scFv16 complex was set as the 3D template for autopicking, and 2,074,844 particles were picked in RELION 3.0^55^. Four cycles of 2D classification were performed. After 2D classification, the particles with good features were further subjected to initial model building and 3D classification. Finally, 4,741,844 particles were further subjected to Bayesian particle polishing and CTF refinement. During postprocessing, different masks were applied on the global refinement map, and a map with a global resolution of 3.33 Å (FSC = 0.143) was obtained. To improve the map quality, the particles were transferred to cryoSPARC for homogeneous and nonuniform refinement. Finally, a density map was obtained with a global resolution of 3.29 Å (FSC = 0.143). Estimation of the local resolution was performed in cryoSPARC. For the ramelteon-MT_2_–G_i_–scFv16 complex, 4609 movies were collected and processed by cryoSPARC v.3.2.0^56^. Beam-induced motion correction was performed using patch motion correction, and contrast transfer function (CTF) parameters were estimated by patch CTF estimation in cryoSPARC. Templates for particle selection were generated by projecting the 3D volume of the ramelteon-MT_1_-G_i_-scFV16 complex. A total of 5,234,455 particles were selected by autopicking (particle diameter set as 150 Å and Lowpass filter as 20 Å) as well as inspection picks, and they were then subjected to 2D classification. Three cycles of 2D classification yielded 847,573 particles with clear features. Six initial models were built by ab initio reconstruction. After that, 639,905 particles based on three 3D classes were selected for homogenous refinement in cryoSPARC. To improve the map quality, local refinement and sharpening were performed using the local refinement calculated B-factor. Finally, a density map was obtained with a global resolution of 3.5 Å (determined by gold standard FSC using the 0.143 criteria). Estimation of the local resolution was performed in cryoSPARC using the volume and mask of the local refinement output.

### Model building, refinement, and computational docking

To build the 2-iodomelatonin-MT_1_-G_i_-scFv16 complex, the crystal structure of 2-iodomelatonin bound MT_1_ (PDB 6ME4) was used as the starting model. To build the ramelteon-MT_1_-G_i_-scFv16 complex, the built 2-iodomelatonin-MT_1_-G_i_ cryo-EM structure and ramelteon-MT_1_ crystal structure (PDB 6ME2) were used as the guiding models. For the ramelteon-MT_2_-G_i_-scFv16 complex, the 2-iodomelatonin-MT_1_-G_i_ and ramelteon-MT_2_ structures (PDB 6ME9) were used as the guiding models. For the G protein in the three complexes, the Gi heterotrimer from the FPR2–Gi cryo-EM structure (PDB 6OMM) was used as the initial model. All the models were first docked into the EM density map using Chimera^57^, followed by iterative manual adjustment and rebuilding in COOT^58^ and phenix.real_space_refine in Phenix^59^. The final model statistics were validated using MolProbity^60^. The molecular graphic figures were prepared with UCSF Chimera, UCSF ChimeraX^61^, and PyMOL. Docking of 5-HEAT and CTL 01-05-B-A05 to melatonin receptors was performed using AutoDock Vina^62^. Receptor or ligand structures were optimized using AutoDockTools-1.5.6. We set a 25×25×35 Å^3^ box as the search region, as such a size was large enough for ligand binding. The outputs of the autodock were analyzed with PyMOL.

### Pocket diameter determination

Channel dimensions were obtained using CAVER analyst v3.0.3^63^. Before performing the calculation, hydrogens were added to the corresponding coordinates using PyMOL. The region around the perceived channel entrance was manually set as the starting point of the tunnel. Using default program parameters, channel dimensions were extracted and trimmed to the segment between the channel entrance and ligand centroid. For visual comparison and comprehension, the section view prepared with PyMOL is shown.

### cAMP assay

G-protein activation was measured using the cAMP-Gi KIT (cisbio, 62AM9PEB). The day before transfection, HEK293 cells (ATCC CRL-1573) were seeded at a density of 0.8 × 10^6^ cells per well in 6-well culture plates and incubated at 37 °C with 5% CO2. Cells were transiently transfected with plasmids coding the wild-type melatonin receptor or mutant receptor using Lipo8000 (Beyotime, C0533). Twenty-four hours posttransfection, the culture media was removed, and the cells were washed with PBS buffer. The transfected HEK293 cells were then plated into white 384-well plates (4000/well) in stimulation buffer and treated with 20 μΜ forskolin, 500 μM IBMX, and test agonist for 30 min. After that, 5 μL cAMP Eu-cryptate reagent and 5 μl anti-cAMP-d2 working solution were added^64^. After incubation at room temperature for 1 h, fluorescence was acquired at 620/665 nm using a FRET reader (PCLARIOstar Plus)^65^ and analyzed with OriginPro 2021. The expression level of each construct was determined by flow cytometry analysis and was used to normalize the cAMP level. Specifically, transfected cells were labeled with DYKDDDDK Tag (D6W5B) Rabbit mAb (cell signaling technology, 15009S), and then washed with PBS. Cells were resuspended in ice-cold PBS, and the fluorescent intensity of single cells was quantified by CytoFLEX (Beckman) flow cytometer equipped with a 488 nm laser. The flow cytometry data were recorded and analyzed with CytExpert 2.3.0.84. Values of mean fluorescence intensity from ~20,000 cells per sample were used for analysis. Statistical analysis was performed with either one-way analysis of variance and a Dunnetts post-test or a paired *t*-test, and significance accepted at *P* < 0.05.

### Reporting summary

Further information on research design is available in the Nature Research Reporting Summary linked to this article.

## Supplementary information

Supplementary Information

Reporting Summary

## Data Availability

The data that support this study are available from the corresponding authors upon reasonable request. The density maps and structure coordinates have been deposited to the Electron Microscopy Database (EMDB) and the protein data bank (PDB) with the following accession codes: EMD-31980 and 7VGY for the 2-iodomelatonin-MT_1_-G_i_-scFv16 complex; EMD-31981 and 7VGZ for the ramelteon-MT_1_-G_i_-scFV16 complex; and EMD-31982 and 7VH0 for the ramelteon-MT_2_-G_i_-scFV16 complex. The PDB datasets used for analysis in this study include 6ME2, 6ME4, 6ME9, 7DB6, 6OMM, 7JVR, 6OSA, 6N4B, 7JJO, 7JV5, and 7AUE).
