# Supplementary material for: Structural basis of the ligand binding and signaling mechanism of melatonin receptors
Source: Nat Commun. 2022 Jan 24;13:454. doi: 10.1038/s41467-022-28111-3 (PMC8786939; doi:10.1038/s41467-022-28111-3)
Supplement: Supplementary file 1 — Supplementary Information [file 41467_2022_28111_MOESM1_ESM.pdf]

Supplementary Information for

Structural basis of the ligand binding and signaling mechanism of melatonin receptors

Qinggong Wang[1,#], Qiuyuan Lu[2,#], Qiong Guo[1], Maikun Teng[3], Qingguo Gong[1], Xu Li[3], Yang Du[2*], Zheng Liu[2*], Yuyong Tao[1*]

[#]These authors contributed equally.

1. Department of Clinical Laboratory, The First Affiliated Hospital of USTC, Ministry of Education Key Laboratory for Membraneless Organelles & Cellular Dynamics, Biomedical Sciences and Health Laboratory of Anhui Province, School of Life Sciences, Division of Life Sciences and Medicine, University of Science and Technology of China, 230027 Hefei, P.R. China.
2. Kobilka Institute of Innovative Drug Discovery, School of Life and Health Sciences, Chinese University of Hong Kong, Shenzhen, Guangdong 518172, China.
3. Ministry of Education Key Laboratory for Membraneless Organelles & Cellular Dynamics, Biomedical Sciences and Health Laboratory of Anhui Province, School of Life Sciences, Division of Life Sciences and Medicine, University of Science and Technology of China, 230027 Hefei, P.R. China.

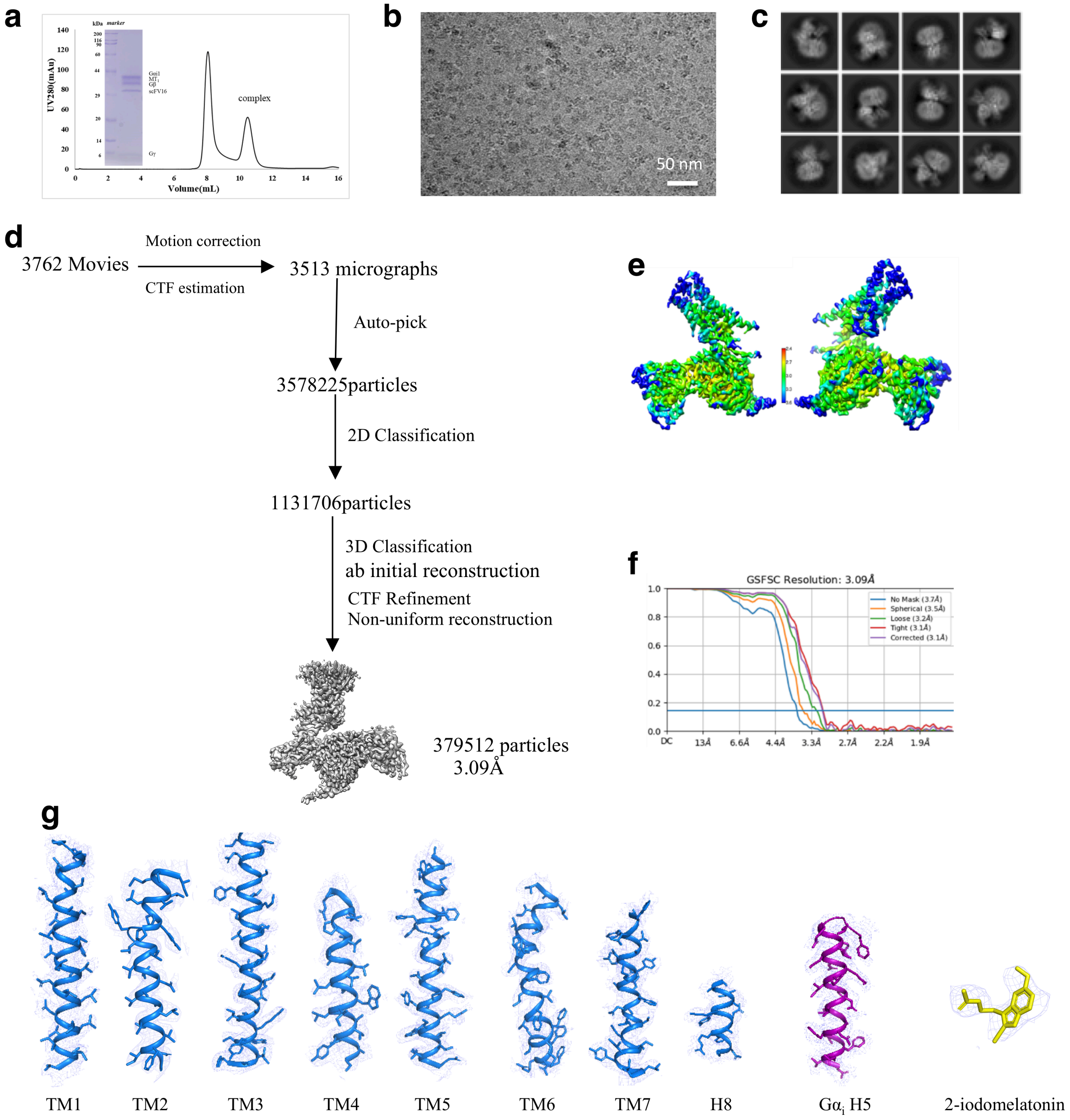

Supplementary Fig. 1: Sample preparation and cryo-EM structure determination of the 2-iodomelatonin-MT$_1$-G$_i$-scFv16 complex.

a Size-exclusion chromatography and coomassie blue stained SDS–PAGE of the purified complex. Experiments were repeated three times with similar results. **b** Representative cryo-EM micrograph of the complex. Four times experiments were repeated independently with similar results. **c** Representative 2D classification. **d** Cryo-EM data processing workflow. **e** Local resolution map of the complex. **f** Gold-standard FSC curves of the 3D reconstructions. **g** Cryo-EM density maps and models of the seven transmembrane helices (TM1-7), Helix-8 (H8), Gαi H5 and 2-iodomelatonin of the complex. Maps are shown in slate, MT$_1$ in marine, Gα$_i$ in purple, 2-iodomelatonin in yellow.

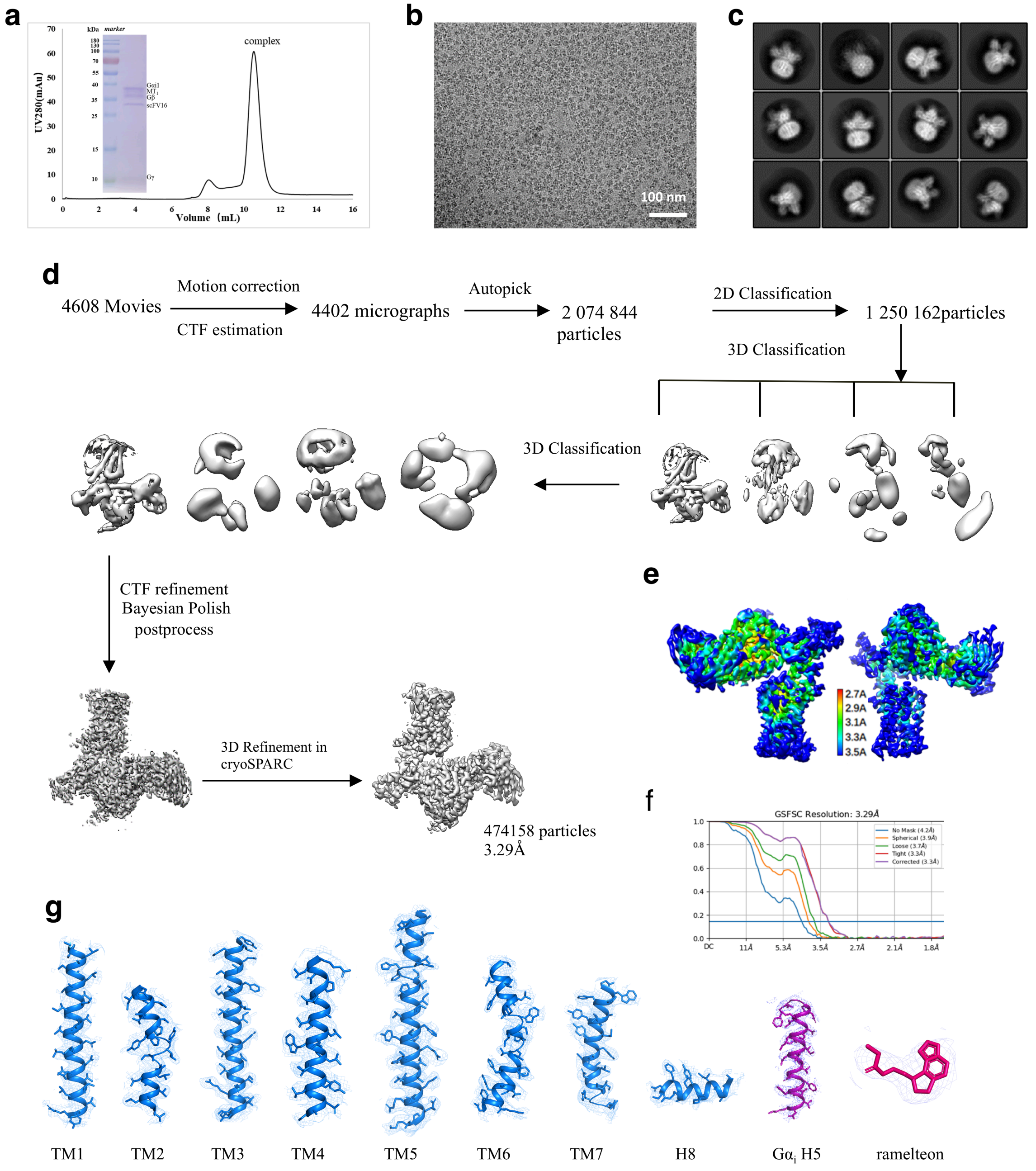

Supplementary Fig. 2: Sample preparation and cryo-EM structure determination of ramelteon-MT$_1$-G$_i$-scFv16 complex.

a Size-exclusion chromatography and coomassie blue stained SDS–PAGE of the complex. Experiments were repeated three times with similar results. **b** Representative cryo-EM micrograph of the complex. Four times experiments were repeated independently with similar results. **c** Representative 2D classification. **d** Cryo-EM data processing workflow. **e** Local resolution map of the complex. **f** Gold-standard FSC curves of the 3D reconstructions. **g** Cryo-EM density maps and models of the seven transmembrane helices (TM1-7), Helix-8 (H8), Gαi H5 and ramelteon of the complex. Maps are shown in cyan, MT$_1$ in marine, Gα$_i$ in purple, ramelteon in hotpink.

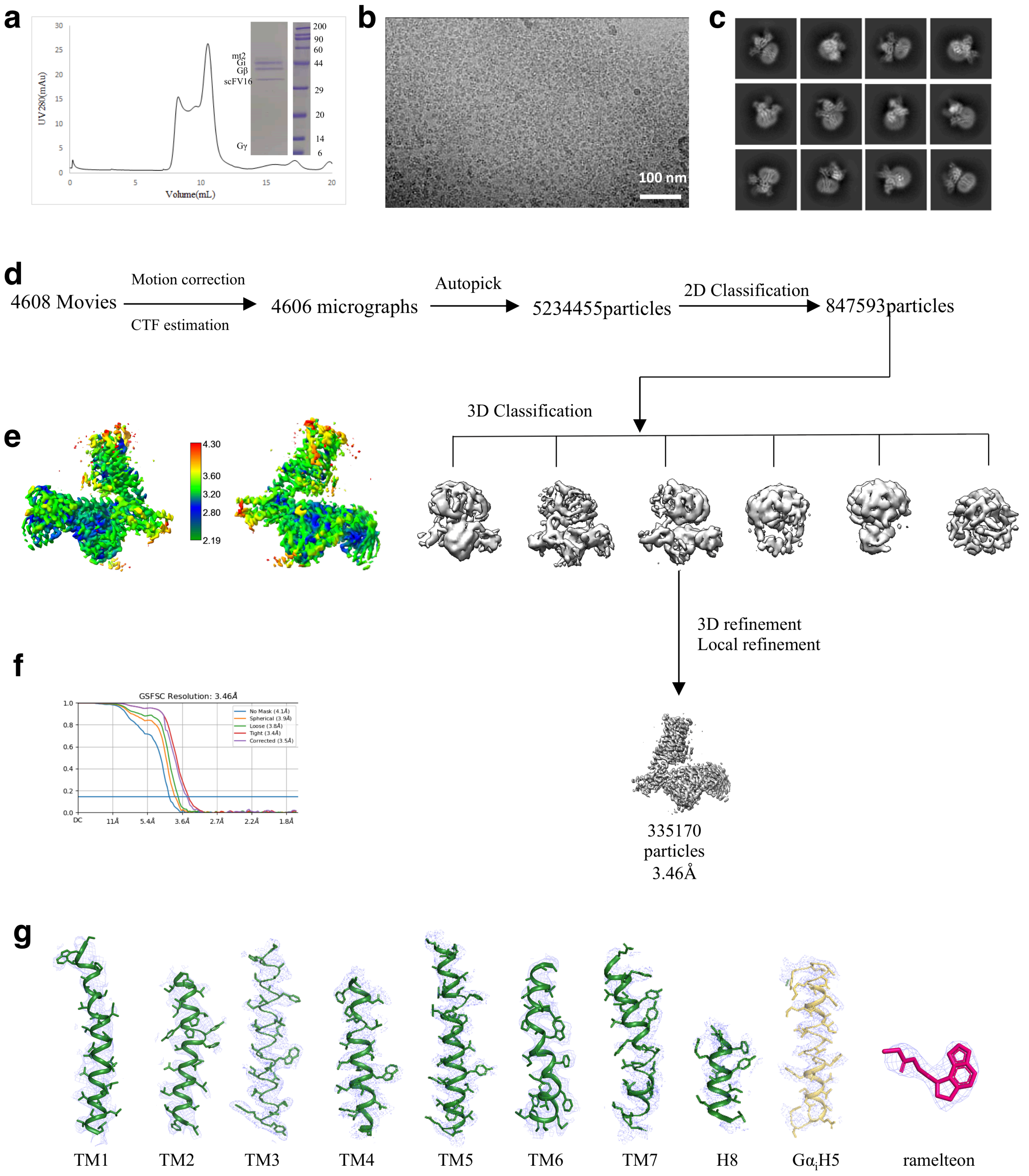

Supplementary Fig. 3: Sample preparation and cryo-EM structure determination of ramelteon-MT$_2$-G$_i$-scFv16 complex.

a Size-exclusion chromatography and coomassie blue stained SDS–PAGE of the purified complex. Experiments were repeated three times with similar results. **b** Representative cryo-EM micrograph of the complex. Four times experiments were repeated independently with similar results. **c** Representative 2D classification. **d** Cryo-EM data processing workflow. **e** Local resolution map of the complex. **f** Gold-standard FSC curves of the 3D reconstructions. **g** Cryo-EM density maps and models of the seven transmembrane helices (TM1-7), Helix-8 (H8), Gαi H5 and ramelteon of the complex. Maps are shown in skyblue, MT$_2$ in green, Gα$_i$ in yellow, ramelteon in hotpink.

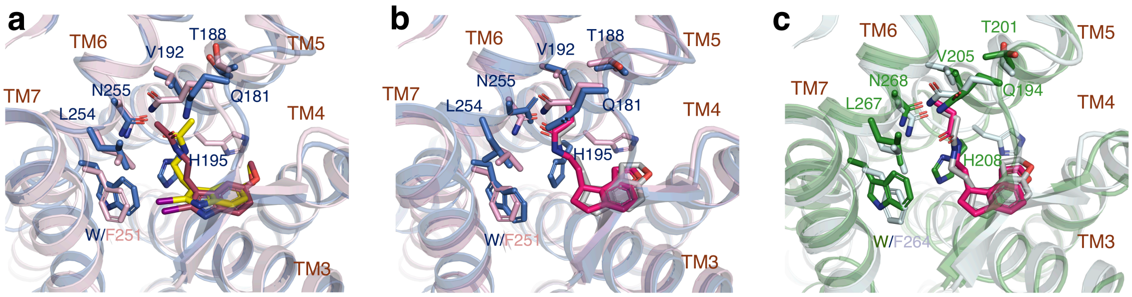

Supplementary Fig. 4: Comparison of the ligand from active and inactive structures.
a 2-iodomelatonin bound in active (yellow) and inactive (brown, PDB ID: 6ME4) MT_1. Active and inactive MT_1 is shown in blue and pink, respectively. **b** Ramelteon bound in active (hotpink) and inactive (gray, PDB ID: 6ME2) MT_1. Active and inactive MT_1 is shown in blue and pink, respectively. **c** Ramelteon bound in active (hotpink) and inactive (gray, PDB ID: 6ME9) MT_2. Active and inactive MT_2 are shown in green and palecyan, respectively.

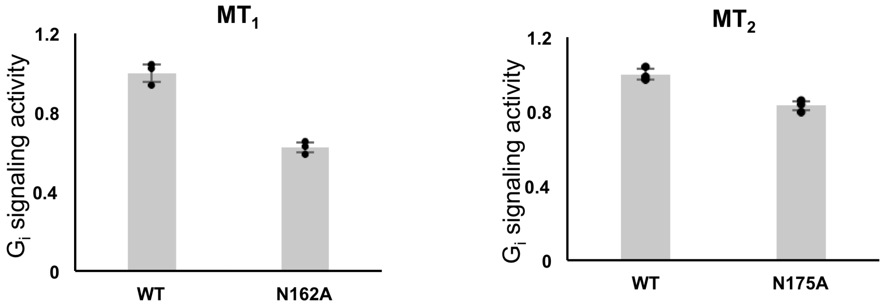

Supplementary Fig. 5: The G_i protein signaling ability of the $N^{4.60}$ mutant of MT_1 and MT_2.
Effects of the $N^{4.60}$ mutation on the G_i signaling ability of MT_1 (left) and MT_2 (right). Data were normalized to that of the wild type receptor and presented as means ± standard error of three replicates.

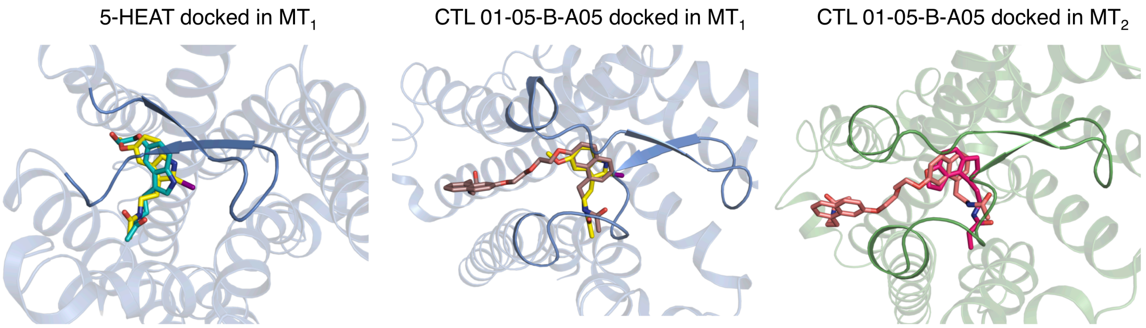

mode	Affinity (kcal/mol)	dist from best mode	
		rmsd l.b.	rmsd u.b
1	-8.6	0	0
2	-7.6	1.977	6.26

mode	Affinity (kcal/mol)	dist from best mode	
		rmsd l.b.	rmsd u.b
1	-9.9	0	0
2	-9.8	2.909	4.663

mode	Affinity (kcal/mol)	dist from best mode	
		rmsd l.b.	rmsd u.b
1	-10.3	0	0
2	-9.3	2.851	11.408

Supplementary Fig. 6: Comparison of the docked 5-HEAT and CTL 01-05-B-A05 with the 2-iodomelatonin and ramelteon bound in MT_1 and MT_2.
5-HEAT (cyan), 2-iodomelatonin (yellow), CTL 01-05-B-A05 (salmon) and ramelteon (pink) are shown as sticks. The docking scores of the first two modes are presented. In addition to the docking score, how the molecules overlay with the known 2-iodomelatonin or ramelteon is another criteria. The mode 2 (highlighted in green) of the two CTL 01-05-B-A05 dockings was chosen, as mode 2 displays better overlap of the first naphthalene group with the 2-iodomelatonin or ramelteon.

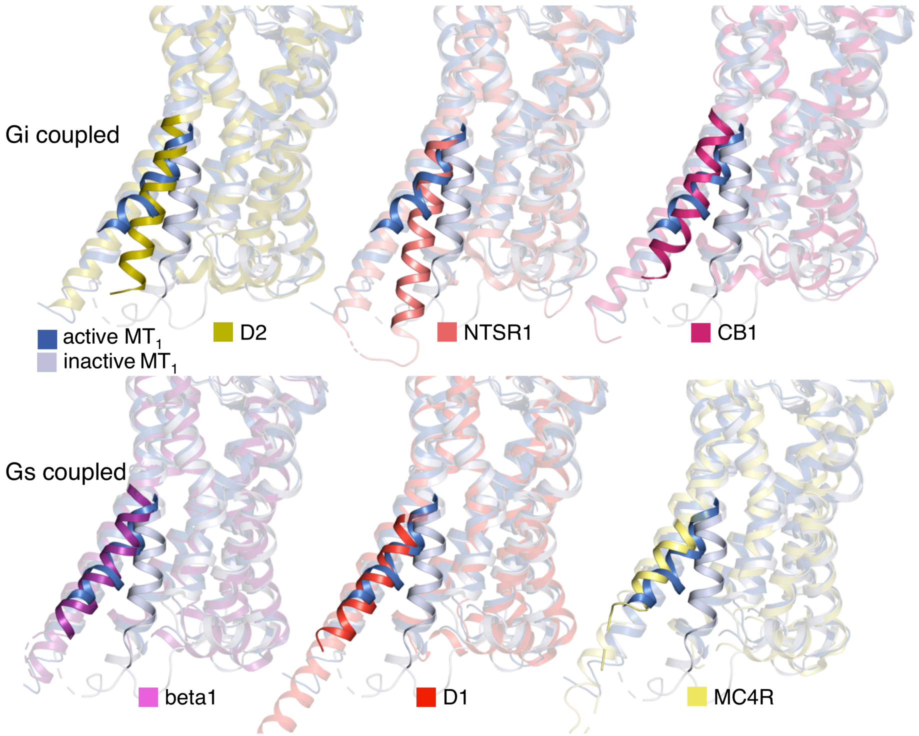

Supplementary Fig. 7: Comparison of the conformation of the TM6 in Gi- (up) and Gs- (below) coupled receptors. The following receptors are used: dopamine receptor 2 (PDB ID:

7JVR), neurotensin receptor 1 (PDB ID: 6OSA), cannabinoid receptor 1 (PDB ID: 6N4B), beta1 adrenergic receptor (PDB ID: 7JJO), dopamine receptor 1 (PDB ID: 7JV5) and melanocortin receptor 4 (PDB ID: 7AUE).

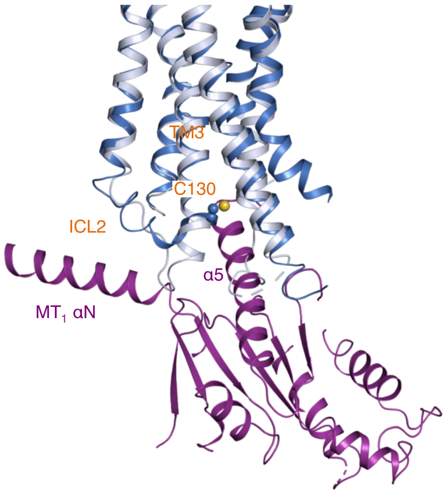

Supplementary Fig. 8: Overlay of the inactive MT$_1$ onto the active MT$_1$-G$_i$ complex.
The ICL2 from the inactive MT$_1$ make clashes with the αN helix in the G$_i$. C130 is shown in spheres. Inactive MT$_1$ is colored in light-blue, active MT$_1$ in marine, G$_i$ in purple.

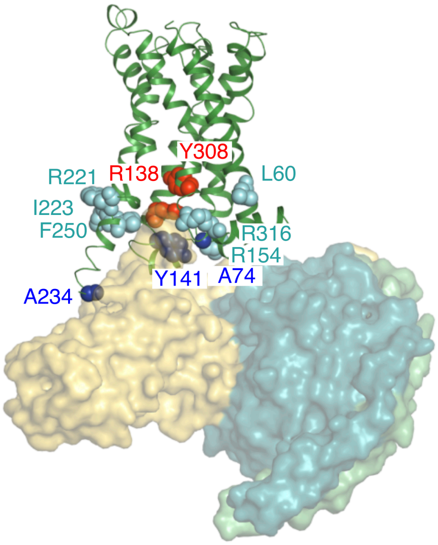

Supplementary Fig. 9: **MT$_2$ mutations related to type 2 diabetes.**
Mutations including A42P, L60R, A74T, P95L, S123R, V124I, R138C, R138H, R138L, Y141F, R154H, T201M, R222H, I223T, F250W, Y308S, R316H, R330W and A342V were reported to impair the G$_i$ signaling ability. Affected sites around the cytoplasmic core are mapped onto the structure. The three classes of the affected residues are colored red, blue and cyan, respectively.

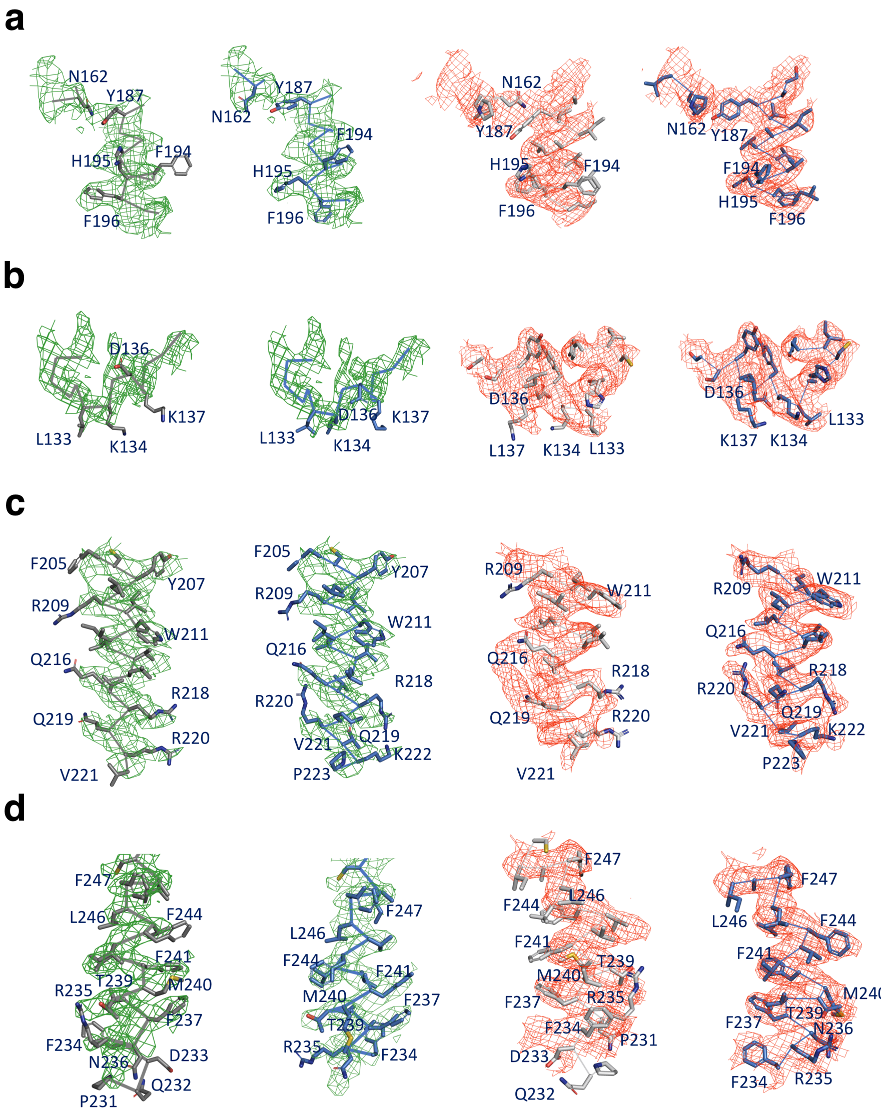

Supplementary Fig. 10: Comparison of the key regions in the two active structures of MT_1. Comparison of the MT_1 N162-Y187-H195 motif **(a)**, ICL2 **(b)**, TM5 **(c)** and TM6 **(d)** from this work and 7DB6. Model and map from 7DB6 are colored in gray and green, respectively. The model and map from this work are colored in blue and red, respectively.

Supplementary Table 1. Statistics for Cryo-EM data collection and processing.

**Data collection and processing**	**ramelteon-MT$_1$-G$_i$-scFv16**	**2-iodomelatonin -MT$_1$-G$_i$-scFv16**	**ramelteon-MT$_2$-G$_i$-scFv16**
Magnification	100500	100500	100500
Voltage (kV)	300	300	300
Electron exposure (e–/Å2)	76.07	76.07	76.07
Defocus range (μm)	-1.2~-2.0	-1.2~-2.0	-1.2~-2.0
Pixel size (Å)	0.83	0.83	0.83
Symmetry imposed	C1	C1	C1
Initial particle projections (no.)	2074844	3578225	5234455
Final particle projections (no.)	474158	379512	335170
Map resolution (Å)	3.29	3.09	3.46
FSC threshold	0.143	0.143	0.143
**Refinement**			
Initial model used (PDB code)	6ME2/6OMM	6ME4/6OMM	6ME9/6OMM
Model resolution (Å)	3.3/3.17	3.2/3.17	3.3/3.17
FSC threshold	0.5	0.5	0.5
Model resolution range (Å)	50-3.3	50-3.1	50-3.5
Map sharpening B factor (Å2)	-120	-110	-175
Model composition			
Non-hydrogen atoms	8790	8748	8716
Protein residues	1125	1121	1119
Ligand	2	2	1
B factors (Å2)			
Protein	106.2	61.46	91.66
Ligand	131.56	82.04	110.76
R.m.s. deviations			
Bond lengths (Å)	0.004	0.004	0.003
Bond angles (°)	0.668	0.756	0.649
Validation			
MolProbity score	1.72	1.98	1.84
Clashs core	10.09	10.55	8.78
Rotamer outliers (%)	0.11	0.32	0.21
Ramachandran plot			
Favored (%)	96.84	93.29	94.58
Allowed (%)	3.16	6.71	5.42
