# Supplementary material for: Structural basis of the ligand binding and signaling mechanism of melatonin receptors
Source: Nat Commun. 2022 Jan 24;13:454. doi: 10.1038/s41467-022-28111-3 (PMC8786939; doi:10.1038/s41467-022-28111-3)
Supplement: Supplementary file 2 — Reporting Summary [file 41467_2022_28111_MOESM2_ESM.pdf]

nature portfolio

Corresponding author(s): Yuyong Tao

Last updated by author(s): Dec 21, 2021

Reporting Summary

Nature Portfolio wishes to improve the reproducibility of the work that we publish. This form provides structure for consistency and transparency in reporting. For further information on Nature Portfolio policies, see our Editorial Policies and the Editorial Policy Checklist.

Statistics

For all statistical analyses, confirm that the following items are present in the figure legend, table legend, main text, or Methods section.

n/a	Confirmed	
☐	☒	The exact sample size (*n*) for each experimental group/condition, given as a discrete number and unit of measurement
☐	☒	A statement on whether measurements were taken from distinct samples or whether the same sample was measured repeatedly
☐	☒	The statistical test(s) used AND whether they are one- or two-sided<br>*Only common tests should be described solely by name; describe more complex techniques in the Methods section.*
☒	☐	A description of all covariates tested
☒	☐	A description of any assumptions or corrections, such as tests of normality and adjustment for multiple comparisons
☐	☒	A full description of the statistical parameters including central tendency (e.g. means) or other basic estimates (e.g. regression coefficient) AND variation (e.g. standard deviation) or associated estimates of uncertainty (e.g. confidence intervals)
☒	☐	For null hypothesis testing, the test statistic (e.g. *F*, *t*, *r*) with confidence intervals, effect sizes, degrees of freedom and *P* value noted<br>*Give P values as exact values whenever suitable.*
☒	☐	For Bayesian analysis, information on the choice of priors and Markov chain Monte Carlo settings
☒	☐	For hierarchical and complex designs, identification of the appropriate level for tests and full reporting of outcomes
☒	☐	Estimates of effect sizes (e.g. Cohen's *d*, Pearson's *r*), indicating how they were calculated

Our web collection on statistics for biologists contains articles on many of the points above.

Software and code

Policy information about availability of computer code

Data collection	cryo-EM data collected on Titan Krios using SerialEM 3.7.
Data analysis	MotionCor2.1, cryoSPRAC 3.2.0, Relion 3.0, Phenix 1.14, COOT 0.8.9, UCSF Chimera 1.12, PyMol 2.0.1, UCSF Chimera X, OriginPro 2021, CAVER 3.0.3, AutoDockTools-1.5.6, CytExpert 2.3.0.84.

For manuscripts utilizing custom algorithms or software that are central to the research but not yet described in published literature, software must be made available to editors and reviewers. We strongly encourage code deposition in a community repository (e.g. GitHub). See the Nature Portfolio guidelines for submitting code & software for further information.

Data

Policy information about availability of data

All manuscripts must include a data availability statement. This statement should provide the following information, where applicable:

- Accession codes, unique identifiers, or web links for publicly available datasets
- A description of any restrictions on data availability
- For clinical datasets or third party data, please ensure that the statement adheres to our policy

The coordinate and cryo-EM map of 2-iodomelatonin-MT1-Gi-scFv16, ramelteon-MT1-Gi-scFv16 and ramelteon-MT2-Gi-scFv16 complex have been deposited to PDB (EMDB) with accession codes 7VGY (EMD-31980), 7VGZ (EMD-31981), 7VH0 (EMD-31982), respectively. The datasets used for analysis in this study includes PDB (6ME2, 6ME4, 6ME9, 7DB6, 6OMM, 7JVR, 6OSA, 6N4B, 7JJO, 7JV5 and 7AUE)

Field-specific reporting

Please select the one below that is the best fit for your research. If you are not sure, read the appropriate sections before making your selection.

☒ Life sciences ☐ Behavioural & social sciences ☐ Ecological, evolutionary & environmental sciences

For a reference copy of the document with all sections, see nature.com/documents/nr-reporting-summary-flat.pdf

Life sciences study design

All studies must disclose on these points even when the disclosure is negative.

Sample size	Sample sizes were not predetermined by statistical methods. For cryo-EM data, images were collected until the resolution and 3D reconstruction converges. For the functional assay, 4000 cells were used for one independent experiment and we use sample size at least of three independent experiments to ensure each data point was repeatable and comparable to other published studies.
Data exclusions	No data were excluded from the analysis.
Replication	Each experiment was repeated at least three times in independent experiments. Experimental findings were reproduced reliably.
Randomization	The cryo-EM data were collected automatically and did not involve choosing. For each datasets of in signaling assays, cells were uniformly seeded in plates before treatment to ensure comparable backgrounds.
Blinding	For cryo-EM study, purified ligand bound-MT-GI complex samples were applied onto a glow-discharged grid and subsequently vitrified using a Vitrobot Mark IV. Cryo-EM imaging was performed on a Titan Krios equipped with a Gatan K3 BioQuantum camera and imaging data were collected automatically. For functional analysis, blinding was not necessary by reason of the quantitative nature of the experiment. All experimental data acquired or analyzed in this study are included in this published article, and subjected to statistical analysis whenever necessary.

Reporting for specific materials, systems and methods

We require information from authors about some types of materials, experimental systems and methods used in many studies. Here, indicate whether each material, system or method listed is relevant to your study. If you are not sure if a list item applies to your research, read the appropriate section before selecting a response.

Materials & experimental systems

n/a	Involved in the study	
☐	☒	Antibodies
☐	☒	Eukaryotic cell lines
☒	☐	Palaeontology and archaeology
☒	☐	Animals and other organisms
☒	☐	Human research participants
☒	☐	Clinical data
☒	☐	Dual use research of concern

Methods

n/a	Involved in the study	
☒	☐	ChIP-seq
☐	☒	Flow cytometry
☒	☐	MRI-based neuroimaging

Antibodies

Antibodies used	DYKDDDDK Tag (D6W5B) Rabbit mAb cell signaling technology, Cat.# 15009S); Anti-flag M1 resin (Sigma–Aldrich Cat# A4596)
Validation	All antibodies were commercially obtained and validation reports are available on the supplier website: https://www.cellsignal.com/products/antibody-conjugates/dykddddk-tag-d6w5b-rabbit-mab-binds-to-same-epitope-as-sigma-s-anti-flag-m2-antibody-alexa-fluor-647-conjugate/15009?_=1637892131886&Ntt=15009s&tahead=true. Anti-flag M1 resin were purchased from sigma-aldrich (https://www.sigmaaldrich.cn/CN/zh/product/sigma/a4596).

Eukaryotic cell lines

Policy information about cell lines

Cell line source(s)	Sf9 (Expression Systems, Cat#94-001F)<br>HEK293 cells (ATCC CRL-1573)
Authentication	No further authentications were performed for this study.

Mycoplasma contamination	No mycoplasma contamination tests were performed for this study.
Commonly misidentified lines (See ICLAC register)	No commonly misidentified cell lines were used.

Flow Cytometry

Plots

Confirm that:

- [x] The axis labels state the marker and fluorochrome used (e.g. CD4-FITC).
- [x] The axis scales are clearly visible. Include numbers along axes only for bottom left plot of group (a 'group' is an analysis of identical markers).
- [x] All plots are contour plots with outliers or pseudocolor plots.
- [x] A numerical value for number of cells or percentage (with statistics) is provided.

Methodology

Sample preparation	HEK-293 cells were washed twice and resuspended in ice-cold PBS after incubation with the substrate.
Instrument	CytoFLEX(Beckman)
Software	CytExpert 2.3.0.84.
Cell population abundance	Approximately 20000 cellular events were collected and the total fluorescence intensity of positive expression cell population was calculated.
Gating strategy	Gating was determined by the Alexa-488 fluorescence intensity to differentiate positive cells and all other cells.

- [x] Tick this box to confirm that a figure exemplifying the gating strategy is provided in the Supplementary Information.
